# Delayed ventricular septal rupture complicating acute inferior wall myocardial infarction

**DOI:** 10.1186/1756-0500-6-124

**Published:** 2013-03-28

**Authors:** Jae Hyung Cho, Srinivasan Sattiraju, Sanjay Mehta, Emil Missov

**Affiliations:** 1 College of Medicine, University of Illinois at Urbana-Champaign, Urbana-Champaign, IL, USA; 2 Division of Cardiology, Carle Heart and Vascular Institute, Carle Foundation Hospital, Urbana-Champaign, IL, USA; 3 Division of Cardiology, University of Minnesota Medical School, 420 Delaware Street SE, Mayo Mail Code 508, Minneapolis, MN, 55455, USA

**Keywords:** Myocardial infarction, Echocardiography, Ventricular septal rupture, Surgery

## Abstract

**Background:**

Ventricular septal rupture is a potentially fatal complication of acute myocardial infarction. Its incidence has declined with modern reperfusion therapy. In the era of percutaneous coronary interventions, it occurs a median of 18–24 hours after myocardial infarction and is most commonly associated with anterior myocardial infarction. We present a case of delayed ventricular septal rupture complicating acute inferior wall myocardial infarction.

**Case presentation:**

A 53-year-old Caucasian male presented with epigastric pain for three days and electrocardiographic evidence for an acute inferior wall myocardial infarction. Coronary angiography revealed a total occlusion of the proximal right coronary artery. Reperfusion was achieved by balloon angioplasty followed by placement of a bare metal stent. On hospital day six, the patient developed acute respiratory distress, a new loud pansystolic murmur, and hemodynamic instability. Echocardiography revealed the presence of a large defect in the inferobasal interventricular septum with significant left-to-right shunt consistent with ventricular septal rupture. The patient underwent emergent surgical repair with a bovine pericardial patch.

**Conclusion:**

Ventricular septal rupture after myocardial infarction should be suspected in the presence of new physical findings and hemodynamic compromise regardless of revascularization therapy.

## Background

Ventricular septal rupture (VSR) is a potentially fatal complication of acute myocardial infarction. Without surgical repair, patients with VSR have an in-hospital mortality rate of 90%. With surgical repair, the in-hospital mortality rate is still 33-45% [1,2]. Before the era of reperfusion therapy, the incidence of VSR was 1-3% [3], declining to 0.2-0.3% with the advent of thrombolytic therapy [4]. In the modern era of ubiquitous percutaneous coronary interventions (PCI), VSR has become a rare finding in patients with acute myocardial infarction. With reperfusion therapy, the median time from the onset of myocardial infarction to the diagnosis of VSR has shortened to 18–24 hours as opposed to 5 days in the pre-reperfusion era [3,5,6]. We present the case of delayed ventricular septal rupture complicating acute inferior wall myocardial infarction.

## Case presentation

**Figure 1:**
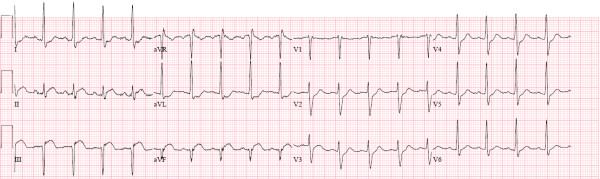
**Electrocardiogram.** Initial ECG showing 2 mm coved ST segment elevation in the inferior leads with Q waves in leads III and aVF, consistent with acute inferior wall myocardial infarction.

**Figure 2:**
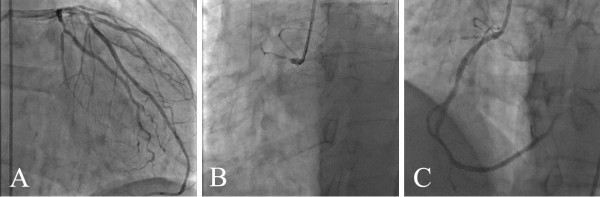
**Coronary angiogram.A**) Left coronary system with diffuse disease. **B**) Thrombotic occlusion of the right coronary artery at the ostium. Note the absence of ipsilateral or contralateral collateral circulation. **C**) Following reperfusion, the right coronary artery shows marginal flow past the bifurcation due to distal embolization. The posterior descending and posterior lateral coronary arteries are faintly visible.

**Figure 3:**
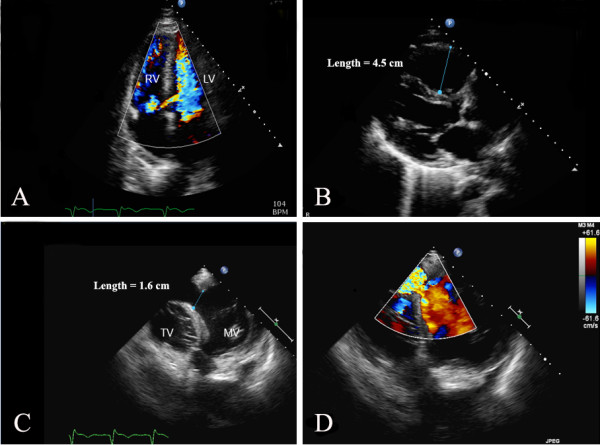
**Echocardiogram.A**) Transthoracic echocardiogram in the apical 4 chamber view showing a ventricular septal rupture in the inferobasal myocardial septum with left-to-right shunt by color Doppler mapping. **B**) Transthoracic echocardiogram in the parasternal long axis view showing significant right ventricular enlargement (proximal right ventricular outflow tract in this image) secondary to left-to-right shunt and volume overload. **C**) Transesophageal echocardiogram from a modified transgastric view at 0° demonstrates a 1.6 cm defect in the inferobasal septum at the level of the mitral valve (MV) and tricuspid valve (TV). **D**) High velocity turbulent flow of left-to-right shunt by color Doppler across the ventricular septal rupture noted in **C**.

A 53-year-old Caucasian male presented to the emergency department with epigastric pain for three days. The electrocardiogram (ECG) showed 2 mm coved ST segment elevation in the inferior leads and Q waves in leads III and aVF suggestive of acute inferior myocardial wall infarction (Figure 1). He underwent emergent coronary angiography which revealed a diffusely diseased left coronary system (Figure 2A) and a total occlusion of the proximal right coronary artery (Figure 2B). Coronary blood flow was successfully restored with balloon angioplasty followed by placement of a bare metal stent (Figure 2C). However, the right coronary artery remained partially occluded after the bifurcation due to distal embolization. Transthoracic echocardiography (TTE) showed akinesis of the basal inferior myocardial segment. On hospital day six, the patient developed the sudden onset of respiratory distress and hemodynamic instability with a new loud pansystolic murmur heard over the entire precordium. Repeat TTE showed a high velocity left-to-right turbulent jet across the inferobasal septum indicative of a VSR (Figure 3A). Dilatation of the right ventricle was also noted (Figure 3B). The patient became tachypneic, tachycardic and hypotensive, and required placement of an intra-aortic balloon pump for hemodynamic support. A transesophageal echocardiogram (TEE) confirmed the presence of a large defect in the inferobasal septum measuring approximately 1.6 cm causing a significant left-to-right shunt consistent with VSR (Figure 3C and D). The patient underwent emergent repair of the VSR with a bovine pericardial patch. His postoperative course was uncomplicated.

## Discussion

Rupture of the ventricular septum is a rare but well recognized complication of acute myocardial infarction. Reperfusion therapy usually prevents extensive myocardial necrosis thus decreasing the incidence of VSR. Without reperfusion, myocardial necrosis develops within 3–5 days [3]. In the SHOCK Trial Registry, the median time from myocardial infarction to VSR was 18 hours (interquartile range, 5 hours to 36 hours) after thrombolytic therapy [6]. However, PCI sometimes can cause reperfusion injury and induce more damage to the infracted myocardium, especially in cases with total occlusion of the infarct-related artery and minimal collateral circulation [5,7]. VSR occurs in the setting of anterior myocardial infarction 70% of the time and complicates inferior myocardial infarction in 29% of the cases [5]. The culprit vessel was the left anterior descending artery in 64% of patients and the right coronary artery in 28% of patients in the GUSTO-I Trial [5]. The location of VSR was apical in 66% and basal in 34% of patients who developed VSR after acute myocardial infarction [4]. Our patient developed symptoms 3 days prior to seeking medical attention and rupture of the inferobasal septum, which is supplied by the right coronary artery occurred six days after revascularization. The late-onset VSR in the setting of acute inferior myocardial infarction is a rare finding in view of reperfusion therapy with PCI. Both TTE and TEE are effective imaging modalities to establish the diagnosis and exclude other mechanical complications of myocardial infarction with similar clinical presentation. The sensitivity and specificity of color Doppler echocardiography for detecting a shunt at the ventricular septal level have been reported as high as 100% [3]. Risk factors for the development of VSR include old age, female gender, anterior myocardial infarction, and single vessel disease, specifically left anterior descending artery [3,5]. The treatment of VSR is emergent surgical repair but even with surgical repair, the in-hospital mortality rate is in the 33-45% range [1,2]. Recently, transcatheter ventricular septal closure has shown promising results in the treatment of VSR with a 30-day mortality rate of 35%. It can be considered as an alternative to surgical repair in hemodynamically stable patients [4]. In our patient, surgery was selected because of hemodynamic instability.

## Conclusion

Although the incidence of VSR has significantly decreased in the modern era of PCI, septal rupture after myocardial infarction should be suspected in the presence of new physical findings and hemodynamic compromise regardless of revascularization therapy.

## Consent

Written informed consent was obtained from the patient for publication of this Case Report and any accompanying images. A copy of the written consent is available for review by the Editor-in-Chief of this journal. Ethical approval is not applicable as this manuscript is not based on experimental research.

## Competing interests

The authors declare that they have no competing interests.

## Authors’ contributions

JHC, SS, SM, and EM contributed to the writing of the manuscript. All authors reviewed and approved the final version of the manuscript.
